# Biological Characteristics of HLA-G and Its Role in Solid Organ Transplantation

**DOI:** 10.3389/fimmu.2022.902093

**Published:** 2022-06-13

**Authors:** Siqi Liu, Nicolaas A. Bos, Erik A. M. Verschuuren, Debbie van Baarle, Johanna Westra

**Affiliations:** ^1^ Department of Rheumatology and Clinical Immunology, University Medical Center Groningen, University of Groningen, Groningen, Netherlands; ^2^ Department of Pulmonary Diseases, University Medical Center Groningen, University of Groningen, Groningen, Netherlands; ^3^ Department of Medical Microbiology and Infection Prevention, University Medical Center Groningen, Groningen, Netherlands

**Keywords:** organ transplantation, HLA-G, rejection, polymorphisms, immunosuppressive treatment, leukocyte immunoglobulin-like receptor, immune regulation

## Abstract

Organ transplantation is a lifesaving option for patients with advanced diseases. Rejection is regarded as one of the most severe risk factors post-transplantation. A molecule that contributes to immune tolerance and resisting rejection is human leukocyte antigen (HLA)-G, which belongs to the non-classical major histocompatibility complex class (MHC) I family. HLA-G was originally found to play a role during pregnancy to maintain immune tolerance between mother and child. It is expressed in the placenta and detected in several body fluids as soluble factor as well as different membrane isoforms on cells. Recent findings on HLA-G show that it can also play multifaceted roles during transplantation. This review will explain the general characteristics and biological function of HLA-G and summarize the views supporting the tolerogenic and other roles of HLA-G to better understand its role in solid organ transplantation (SOT) and its complications. Finally, we will discuss potential future research on the role of HLA-G in prevention, diagnosis, and treatment in SOT.

## Introduction

Solid organ transplantation (SOT) is a therapeutic option for terminal stage organ dysfunction of the heart, liver, kidney, pancreas, small bowel, and lung intending to prolong the quality of life and life expectation (1). Rejection is one of the most severe complications of SOT. Rejection occurs when donor tissues are not recognized as self by the recipient’s immune system, which will trigger an inflammatory response leading to rejection of the transplanted tissues. Rejection can occur at different moments after transplantation. The highest risk for rejection lies during the first months, but rejection can also occur at a later stage. Acute rejection often occurs within 6 months after transplantation. The mechanism of acute rejection involves specific lymphocytes that react to the non-self-human leukocyte antigens from the graft. Chronic rejection occurs months to years after transplantation. Acute rejection is often a risk factor for development of chronic rejection. Chronic rejection involves the formation of matrix proteins such as collagen by smooth muscle cells resulting in graft arteriosclerosis and fibrosis. To treat rejection, immunosuppressive medication is used which targets T cell responses but as a side effect severely impairs the general immunity (2). Therefore SOT patients are at high risk for infections. Subsequently, for the treatment of SOT patients, a balance between the risk of rejection and the level of immunosuppression needs to be found.

Immune regulation is important in maintaining this balance and is relevant to lowering the chance of rejection (3). HLA-G is a regulatory molecule, first described in fetal cytotrophoblasts during pregnancy, and is thought to play a role in protecting the fetus from destruction by the mother’s immune system, so-called fetal-maternal tolerance (4). In physiological conditions, the molecule is expressed in extra villous trophoblasts cells (5), but also in other tissues. In pathological situations, the molecule is observed in tumors (6) and correlated to bad prognosis, and is associated to autoimmune diseases and viral infection susceptibility, creating an unbalanced and pathologic environment (7, 8). HLA-G has seven isoforms and has different polymorphisms and biological characteristics, which leads to different expression levels. Its inhibitory function is conveyed through the interaction with specific receptors on a diverse set of immune cells. HLA-G is thought to be involved in transplantation immune tolerance by protecting the organ from rejection (9).

This review aims to introduce the biologic characteristics of HLA-G, to summarize the views supporting the tolerogenic and other roles of HLA-G, and to discuss its potential role in protecting the transplanted organ from rejection. Finally we discuss potential future research on the role of HLA-G in prevention, diagnosis, and treatment in SOT.

## HLA-G Biology Characteristics

### The HLA-G Gene and Polymorphisms

The HLA-G gene was first referred to as a member of the non-classical MHC I family in 1987 (10). It is located in the MHC region at chromosome 6p21.3 (11). The non-classical MHC-I has fewer polymorphisms and has limited tissue distribution compared to classical MHC-I (12). Classical MHC-I has an important role in adaptive immunity by antigen-presentation to CD8+T cells. Non-classical MHC-I is important in the regulation of both adaptive and innate immune responses (13).

**Figure 1:**
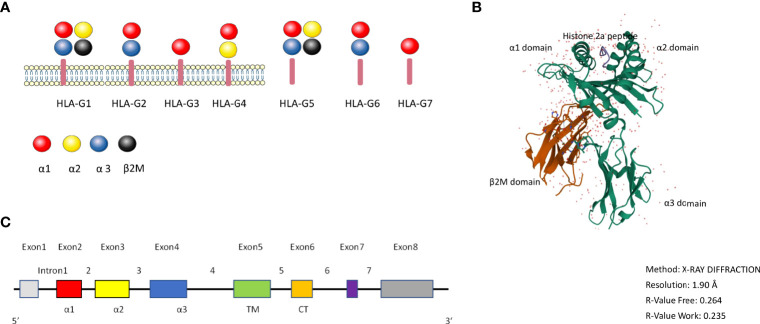
the HLA-G molecule. **(A)**: Schematic overview of the HLA-G isoforms. HLA-G1 to G4 are membrane bound isoforms and HLA-G5 to G7 are soluble isoforms, they are generated by alternative splicing. HLA-G1 and G5 complex contain α1 (red color), α2 (yellow color), and α3 (blue color) globular domains non-covalently associated with β2-microglobulin (black color). **(B)**: 3D crystal structure of HLA-G (reproduced from Protein Data Bank (Gene ID: 1YDP) with permission. The heavy chain (hc) is shown in green, β2M in red, and the peptide in blue color. **(C)**: HLA-G gene structure. Exon 1 encodes the signal peptide. Exons 2, 3, and 4 encode the α1, α2, and α3 domains, respectively. Exons 5 and 6 for the transmembrane (TM) and cytoplasmic (CT) domains, respectively. Exon 7 and exon 8 are not translated.

HLA-G has 88 described alleles and is 3151 base pairs in length (14). HLA-G has seven isoforms: it can be expressed as the membrane bound isoforms HLA-G1 (complete molecule and full length protein), HLA-G2 (minus globular α2 domain), HLA-G3 (minus α2 α3 domains), HLA-G4 (minus α3 domain) and the soluble isoforms HLA-G5 (soluble HLA-G1), HLA-G6 (soluble HLA-G2) and HLA-G7 (soluble HLA-G3) (17–39 kDa), which all have immune tolerating properties (15) (**Figure 1A**). The different membrane and soluble isoforms are generated through alternative splicing (16, 17). Compared with other isoforms, HLA-G1 and G5 molecules are more like classical HLA class I antigens (**Figure 1A**). Tronik-Le Roux and colleagues detected a novel HLA-G isoform in clear cell renal cell carcinoma cells, that has an extended 5’-region and lacks the transmembrane and α1 domains (18).

**Figure 1B** shows the 3D crystal structure of HLA-G1 protein derived from the protein data bank website (PDB) (PDB ID: 1YDP), originally shown by Clements et al. (19). Similarly to MHC Class I, it has a heavy chain (hc) with three globular domains α1–α2–α3 which are non-covalently bound to β2-microglobulin (β2M) and a peptide (**Figure 1**). HLA-G1 and HLA-G5 have extensively been studied over the years, because of their similarity to classical MHC-I. Like MHC-I, HLA-G1 and G5 contain α1, α2, and α3 that can combine to β2M, while HLA-G1 and G5 molecules’ α1, α2 domains can form the antigen presenting peptide binding cleft. HLA-G can exist as dimers or as monomers. The dimers modality are linked by disulfide bonds with two cysteine residues at position 42 on the HLA-G α1 domain (20).

Full-length HLA-G has eight exons and seven introns (**Figure 1C**). Exon 1 encodes the signal peptide, exons 2, 3, and 4 encode the α1, α2, and α3 domains, respectively. Exon 5 encodes the transmembrane domain. The appearance of a stop codon at the second codon of exon 6 leads to a HLA-G shortened cytoplasmic tail, and as a result exon 7 and exon 8 are not translated (21). Soluble HLA-G5 and G6 are formed by the stop sequence in intron 4 (22, 23).

There are two noncoding regions in the HLA-G gene: the 5’ upstream regulatory region (URR) and 3’ untranslated regions (UTR), which have the most polymorphic sites. The 3’ UTR polymorphisms are listed in **Table S1**. HLA-G has eight UTR haplotypes and has the capacity to exist in different polymorphisms. Castelli et al’s work based on the 1000 genomes project found that UTR-1 to UTR-8 are the more frequent in the 3’UTRs but not the only 3’UTRs identified (24). Specific polymorphisms such as UTR-1 have been related to recurrent pregnancy loss and other pregnancy disorders (25, 26).

HLA-G*0105N is known as a null allele and has a cytosine deletion in exon 3 leading to a premature stop codon in exon 4 (27, 28). HLA-G*01013 and HLA-G*0105N have been related to low HLA-G protein expression while G*01041 is related to high expression (29). In an *in vitro* study it was found that HLA-G*0105N encoded proteins could protect natural killer (NK) cells from lysis which indicates that this is a functional HLA-G isoform (30). The HLA-G*010101 was the first allele sequenced and was found to be predominantly present in almost all populations (Asian, European, and African) (27).

### Regulation of HLA-G Protein Expression

During pregnancy HLA-G is primarily expressed by extravillous trophoblast cells in the placenta at the fetal maternal interface, which is important in maternal tolerance by inhibition of NK cell lysis (5, 31). HLA-G also is an important player in preeclamptic patients (32), and in patients with recurrent miscarriage (33). Expression of HLA-G in the male reproductive system has also been described (34, 35).

Next to its role during pregnancy soluble HLA-G (sHLA-G) can also be found in body plasma (36), cerebrospinal fluid (37), and even as part of extracellular vesicles (38). A study by Rebman et al. showed that peripheral blood monocytes are the predominant cells secreting HLA-G5 (39). In pathological situations the molecule can be observed in solid tumors (6, 40), in malignant melanocytic lesions (41), malignant ascites (42) and pleural effusions (40). As mentioned, HLA-G expression in malignancy is related to bad prognosis.

Furthermore, membrane bound HLA-G can be detected in peripheral blood on different immune cells such as monocytes/macrophages, regulatory T cells, CD4+ T cells, CD8+ cytotoxic T cells and dendritic cells and may be implicated in the complex mechanisms underlying the pathogenesis of these disorders as described in the review by Contini et al. (7). In general, increased expression of HLA-G may reflect an attempt to control the immune derangement as for instance seen in autoimmune diseases.

HLA-G expression is influenced by genotype and polymorphisms. In general, haplotype UTR-1 is associated with increased sHLA-G levels and UTR-5 or UTR-7 are associated with decreased sHLA-G levels, while for UTRs 2, 3, 4, 6, 8 or 10 no significant differences were found regarding sHLA-G expression (43). The influence of UTR-5 on gene expression show some ambiguous results, since it was associated with both high and low expression of sHLA-G as mentioned in the review by Dahl et al. (44). A possible explanation to this contradictory result is that UTR-5 haplotype may vary among different populations. The variable polymorphisms 14 bp, +3142 C/G and +3187A/G in UTR-3 can influence HLA-G expression by modifying its mRNA stability (27, 45). In general, the HLA-G 14 bp Ins/del, +3142 SNP and +3187A/G seemed to have the most influence on HLA-G expression level.

Next to this, HLA-G expression is also regulated by cytokines. In line with its tolerogenic role there is evidence that anti-inflammatory and immunosuppressive cytokines like Interleukin (IL)-10 and IL-4 can upregulate HLA-G expression on human PBMCs (46). There is enhanced HLA-G expression in trophoblast cells when activated by IL-10 and this suggests that IL-10 may have a role in protecting the semi-allogenic human fetus from maternal immune responses (47). Pro-inflammatory cytokines such as IL-2 and IL-6 are described to down regulate HLA-G expression on the choriocarcinoma cell line JEG-3, which is widely used as an *in vitro* model study of human trophoblast cells (48). This study also showed that IFN-γ and IL-10 have a role in maintaining high expression of HLA-G and regulation of HLA-G isoforms, respectively (48). However, these results have not been shown *in vivo* yet.

### HLA-G Receptors and Mechanisms of Suppression/Regulation

#### HLA-G Receptors

**Figure 2:**
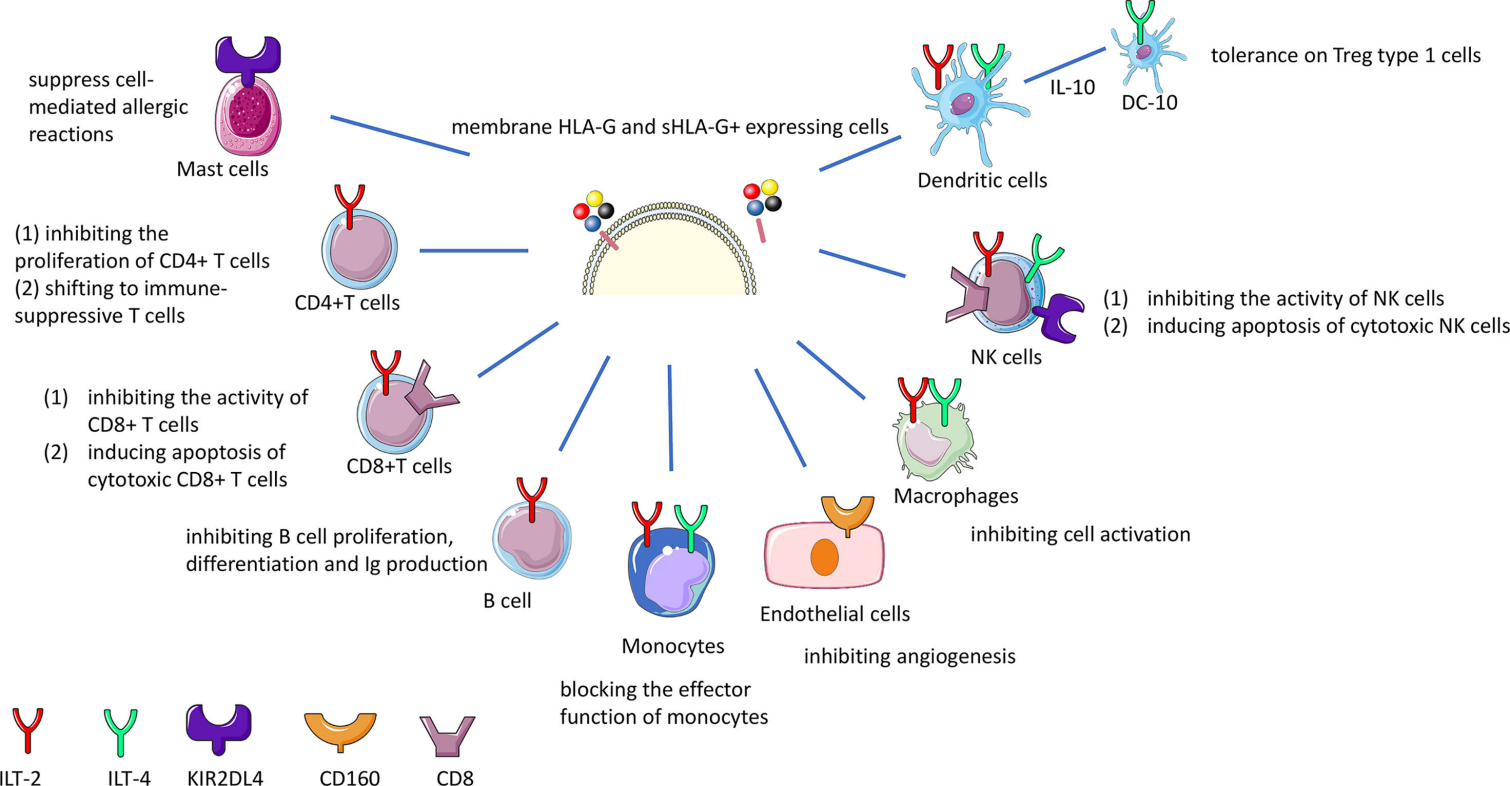
HLA-G immune inhibition by interaction with receptors on effector cells. sHLA-G and membrane bound HLA-G molecules interact with the ILT-2 and ILT-4 receptor on T, NK, B cells and macrophages resulting in the inhibition of cytotoxicity, proliferation, or antibody production. The interaction of HLA-G with CD8 coreceptor on certain T and NK cell population leads to the deletion of these cells. Long-term tolerance will be achieved by the induction of different types of regulatory T (Treg) cells. HLA-G and KIR2DL4 interaction on mast cells suppresses mediation of allergic reactions.

HLA-G interacts with receptors such as Ig-like transcript (ILT)-2 expressed on T cells, B cells, monocytes, macrophages, NK cells and dendritic cells; ILT-4 on monocytes, macrophages, NK cells and dendritic cells; killer cell Ig-like receptor (KIR)2DL4 on mast and NK cells; CD8 on T cells and NK cells, and CD160 on endothelial cells (49–52). In general, interaction with these receptors leads to inhibition of proliferation, of differentiation, of production of cytokines and inhibition of other mechanisms (**Table S2** and **Figure 2**). HLA-G dimers have a higher affinity with the ILT receptors than the monomers (20). ILT-2, ILT-4 and CD8 inhibitory receptors can all bind to sHLA-G *via* the HLA-G α3 domain (53). ILT-2 and ILT-4 can recognize β2-microglobulin, but only ILT-4 can recognize HLA-G free heavy chains (54, 55) and the isoforms HLA-G2 and G6 α1–α3 (56).

ILT-2 (also known as CD85j and LILRB1) is a receptor that consists of 4 structural domains (D1-4), and is the main ligand for the HLA-G dimer, since HLA-G dimers have greater avidity for ILT-2 than ILT-4 (57). HLA-G inhibiting receptor ILT-4 (also known as CD85d and LILRB2) can regulate HLA-G function in dendritic cells, as ILT-4 expression is limited to monocytes, macrophages and dendritic cells (58–60). Interferon (IFN)-γ can induce expression of ILT4 and other HLA-G inhibitory receptor molecules, thereby indirectly acting on HLA-G expression (48, 61). Since ILT-2 and ILT-4 are known inhibitors of the immune system, understanding its regulation by HLA-G, is relevant in the context of organ transplantation disease (see below).

The KIR2DL4 (CD158d) receptor which belongs to the KIR receptors is induced on decidual NK cells or on systemic NK cells (62–64). However, one study showed that binding of KIR2DL4 to HLA-G monomers and dimers was absent (65). Therefore the interaction between HLA-G and KIR2DL4 is not clear and may need further investigation.

Altogether, HLA-G can upregulate the ILT2, ILT4 and KIR2DL4 expression in antigen-presenting cells, NK cells, and T cells (66–68). HLA-G and cells expressing its receptors were found to be related, suggesting an autocrine or paracrine regulation mechanism. These inhibitory receptors show more affinity for HLA-G than the classical HLA class I molecules, suggesting that HLA-G is important in regulating NK cell, T cell, and myelomonocytic cell activation.

CD8 is a receptor for MHC-class I molecules, which can induce apoptosis under certain conditions such as sHLA-G concentrations below 100 ng/ml, or *in vitro* when cells secrete HLA-G5, but this still needs further confirmation in a physiological context (69). HLA-G may increase the expression and secretion of FasL and may induce apoptosis of CD8+ T cells through the Fas–FasL mediated mechanism (39, 47).

HLA-G-CD160 interaction has a direct inhibitory role on vessel formation (70). Also, the interaction between sHLA-G and CD160 on endothelial cells was shown to cause apoptosis. Because CD160 is very often strongly expressed in the vasculature of tumors, future research is recommended to explore the interaction of HLA-G and CD160 in different tumors.

In addition, NKG2A/CD94 was found as a possible new receptor for HLA-G on NK cells, but this needs further research to better understand the role of binding of HLA-G to this receptor (71).

### HLA-G Direct Suppressive Effects on Immune Cells

The HLA-G immunomodulatory role is based on the inhibitory effect on T cells and NK cells through the above mentioned receptors by inhibition of proliferation and cytotoxic functions, regulatory T cell generation, differentiation of antigen-presenting cells (APC), and cytokine secretion. During these processes, HLA-G enhances the expression of Th2 anti-inflammatory cytokines, including IL-4, IL-10, and IL-13, and decreases Th1 pro-inflammatory cytokines, including IL- 2, TNF-α, and IFN-γ.

A flow cytometry study found that HLA-G can exert inhibition of proliferation of CD4+ T cells (72). In a mixed lymphocyte cultures experiment, it was found that sHLA-G5 from the responder CD4+ T cells can inhibit the allogeneic proliferative T cells response (73). It was also shown that HLA-G expression on APCs could inhibit CD4+ T cell activation directly (74–76).

During pregnancy HLA-G may play a critical role in regulating CD8+ T cell function by eliminating alloreactive T cells (23).

Studies have demonstrated that HLA-G can inhibit migration, proliferation, lytic activity of NK cells, production of IFN-γ or other NK T cells cytokines through binding to ILT-2 and KIR2DL4 receptors (17, 49, 71, 77–79).

A mechanistic study showed that dendritic cells (DCs) exposed to IFN-γ have low expression of costimulatory molecules and enhanced expression of HLA-G and ILT-4 molecules (61). Another mechanism is the upregulation of ILT-4 receptors on DCs by CD8+CD28+ Tregs, without inducing apoptosis or maturation of DCs (80). DC-10 is a new subset of DCs characterized by IL-10 production, which can express membrane bound HLA-G when differentiated and thereby induce T regulatory type 1(Tr1) cells (81).

## Transplantation

### HLA-G in Solid Organ Transplantation

Since it was found that HLA-G has a role in protecting the fetus from attack by the maternal immune system, similar functions can be envisioned for allogeneic organ transplantation (82, 83). Acceptance of paternal antigens on the fetus during pregnancy can be considered as a successful allograft in the maternal host. Transplantation can be regarded as a similar allograft placement. Therefore, it is hypothesized that HLA-G has a tolerogenic role which can diminish the risk of rejection and can help to improve the survival of the allograft.

HLA-G expression was first investigated in heart transplantation in 2000 (9). Since then, its expression was detected in different solid organs after transplantation such as in heart, kidney, liver, lung, liver-kidney, kidney-pancreas transplantations. HLA-G was detected using immunohistochemistry in tissue biopsies or by ELISA in serum or plasma, by flow cytometry on specific cell subsets and by real-time PCR on isolated DNA and RNA. As shown in **Table 1**, most of the clinical studies showed that tissue or blood expression of HLA-G with any of the above mentioned techniques has a protective role, induction of immune tolerance and subsequent graft acceptance in transplantation. In some studies however, the relation with graft acceptance was not so clear and in these studies expression of HLA-G was determined by time after transplantation (96), inflammatory processes, or specific genotypes associated with diabetes (110, 123) (see **Table 1**). The study by Moroso et al. for instance showed that HLA-G did not have a protective role after liver transplantation, but that end-stage liver disease was associated with HLA-G expression on hepatocytes (116). The study by White et al. (111) showed contrary to their expectations that increased soluble HLA-G concentrations in bronchoalveolar lavage but not in serum was associated with a higher grade of AR prior to a clinical diagnosis of BOS.

**Table 1:** The association between HLA-G expression and organ transplantation graft acceptance in clinical studies.

Organ	HLA protein expression on graft tissue	(s)HLA-G in blood(mRNA or protein)	HLA genotypes and gene expression	Graft Acceptance	Reference
Heart	Endomyocardial cells (IHC)	sHLA-G5 and -G6 (IP and WB)	ND	Yes	(9, 84)
	ND	sHLA-G (ELISA)	ND	Yes	(83, 85)
	Endomyocardial cells Myocardial biopsies (IHC)	ND	ND	Yes	(86)
	ND	sHLA-G (ELISA),	HLA-G 14bp ins/del (PCR) -14bp/-14bp is related to higher sHLA-G	Yes	(87, 88)
	ND	ND	HLA-G +3196 polymorphism (PCR) is risk factor for cell-mediated rejection	ND	(89)
Kidney	Glomerular and tubular epithelial cells(IHC)	ND	ND	Yes	(90)
	ND	HLA-G mRNA (semiq. PCR)		Yes	(91)
	ND	ND	HLA-G 14bp ins/del (PCR) is related to post-Tx weight gain and complications	No	(92, 93)
	ND	ND	HLA-G 14bp ins/del (PCR) -14bp/-14bp is related to stable disease	Yes	(94, 95)
	ND	sHLA-G (ELISA)	ND	No	(94)
				Yes	(95)
	ND	ND	HLA-G 3’UTR region (DNA sequencing) several associations among different polymorphic sites	ND	(96, 97)
	ND	sHLA-G (ELISA)	HLA-G 14bp ins/del (PCR) -14bp/-14bp is related to CKD	Yes	(98)
	ND	sHLA-G (ELISA	HLA-G 14bp ins/del (PCR) Donor HLA-G polymorphism important for AR	Yes	(99)
	ND	sHLA-G (ELISA)	HLA-G 14bp ins/del (PCR) Higher AR in +14bp/+14bp, higher s-HLA-G in non-AR	Yes	(100)
	ND	sHLA-G (ELISA) HLA-G mRNA (rt- PCR)	5’UTR and 3’UTR (PCR) UTR-haplotypes are involved in different HLA-G expression patterns at transcriptional and translational levels	Yes	(101)
	ND	sHLA-G (ELISA kit) (IP and WB)(Flow Cytometry*)*	ND	Yes	(102)
	ND	sHLA-G1 and G5 (ELISA) HLA-G mRNA (RT-PCR)	ND	Yes	(103)
	tubular epithelial cells (IHC)	HLA-G m RNA (RT-PCR)	ND	ND	(104)
	ND	sHLA-G1 and G5 (ELISA)	HLA-G 3’UTR region (PCR) higher sHLA-G in homozygous +3010GG, +3142CC, +3187GG, and +3196CC carriers in non-AR patients	Yes	(105)
	ND	sHLA-G1 and G5 (ELISA)	HLA-G 3’UTR region (PCR) No relation between sHLA-G levels and genotypes, relation between HLA-G 14bp ins/ins and +3142G/G and obesity and diabetes mellitus post-transplant	No	(106)
	ND	sHLA-G1 and G5 (ELISA)	HLA-G 3’UTR region (PCR) several associations among different polymorphic sites	ND	(107)
	ND	sHLA-G (ELISA)	HLA-G +3142 C>G SNP (PCR) relation to CMV infection	ND	(108)
	tubular epithelial cells (pTECs) (IF)	sHLA-G1 and G5 (ELISA)	ND	Yes	(109)
Lung	bronchial epithelial cells (IHC)	sHLA-G *in vitro* (ELISA) mRNA *in vitro* (RT-PCR)	ND	Yes	(110)
	ND	sHLA-G1 and G5 in plasma and BAL (ELISA)	ND	No	(111)
	bronchiolar and bronchial epithelial cells (IHC)	sHLA-G in plasma ELISA)	ND	Yes	(31)
	Transbronchial biopsies (IF)	sHLA-G in BAL (ELISA)	Donor HLA-G SNPs (PCR) Specific donor SNPs are associated with mortality risk after lung transplantation, while certain donor-recipient SNP-pairings modulated CLAD risk	No	(112)
Liver	ND	sHLA-G (ELISA)(flow cytometry)	ND	Yes	(113)
	ND	sHLA-G1 and G5 (ELISA)	ND	Yes	(114)
	Liver tissues (IHC)	sHLA-G (ELISA)	ND	Yes	(115)
				No	(116)
	ND	ND	HLA-G 14-bp ins/del (PCR) no relations between 14-bp ins/del and acute rejection	No	(117)
	ND	ND	HLA-G 14-bp ins/del (PCR), 3142C>G SNP: 14-bp ins/ins and +3142GG genotypes are of importance AR prediction	Yes	(118)
Kidney, liver and kidney liver combined	Biliary epithelial cells tubular epithelial cells(IHC)	sHLA-G1 and G5 (ELISA)	ND	Yes	(119)
	Biliary epithelial cells mononuclear cells glomerular cells tubular epithelial cells(IHC)	sHLA-G1 and G5 (ELISA)	ND	Yes	(120)
	ND	sHLA-G (ELISA)HLA-G1 and G5 mRNA (RT-PCR)	ND	Yes	(74)
	ND	sHLA-G1 and G5 (ELISA) mRNA(RT-PCR)	ND	Yes	(121)
Kidney kidney/pancreas	ND	sHLA-G (ELISA)	ND	Yes	(122)
Pancreas	ND	ND	HLA-G 14-bp ins/del (Genotyping) 14bp ins/ins genotype is risk factor for susceptibility to type 1 diabetes mellitus	No	(123)

ND, not determined.

Since specific polymorphisms influence HLA-G expression, these polymorphisms can be associated with rejection or acceptance. In most studies genomic DNA of the recipient is used so studies are looking at recipient HLA-G polymorphisms. HLA-G 14bp ins/del and 3142 SNP are most strongly associated with rejection (**Table 1**). The +3003C variant specific for UTR-4 provides a protective role in rejection while the +3196G variant specific for UTR-2 promotes rejection (97). Especially in kidney transplant studies, HLA-G UTR-2, 3, 4, 6 polymorphisms were found more frequently in acute rejection groups while UTR-1, 5, 7 expression was seen mostly in stable cohorts (105).

A study by Lazarte et al. (112) investigated the association of donor and recipient HLA-G SNPs with chronic lung allograft dysfunction (CLAD) and mortality after lung transplantation. Specific donor SNPs were associated with mortality risk after lung transplantation, while certain donor-recipient SNP pairings modulated CLAD risk. Janssen et al. also investigated both kidney transplant recipient- and donor- HLA-G -14bp ins/del and 3142C > G polymorphisms (99). A higher frequency of these genotypes in the donors was seen in no-rejection patients, so these genotypes are protective against transplant rejection. These studies shows that it is relevant whether donor and/or recipient HLA genotypes were determined. While donor genotypes could influence the local HLA-G expression in the transplanted organ, recipient genotypes will likely represent the activity of the host immune system.

### Inhibitory and Regulatory Effects of HLA-G on Immune Cells After Transplantation

As shown above, both HLA-G expression in grafts and sHLA-G levels have often been shown to be associated with improved graft acceptance. T cells exert an important tolerogenic role in organ transplantation, but the relation between T cell function and HLA-G expression is not completely clear. Some studies have shown that HLA-G molecules and HLA-G+ T cells are associated with acceptance in heart and kidney transplantation (84, 124). In 66 kidney transplantation patients measuring HLA-G expression by flow cytometry on CD4+ T cells, it was found that decreasing number of CD4+HLA-G+ cells compared to stable patients could predict AR (125).

Data suggested that occurrence of T follicular helper cells (Tfhs) is related to acute rejection after kidney transplantation (126). However, a single center study with 42 kidney transplantation patients showed no difference in HLA-G expression on Tfhs derived from extracellular vesicles released by cells between rejection and non-rejection groups, suggesting that Tfhs contribute little to allograft transplantation (127).

In human heart transplant recipients, it was shown that CD8+CD28- alloantigen-specific suppressor T cells were associated with non-rejections by inducing upregulation of ILT4 on monocytes and dendritic cells, which suggest that the mechanism of tolerance maybe related to engagement of HLA-G to the ILT4 receptor (59).

DCs have been reported to express high levels of HLA-G in peripheral blood in tolerant liver transplant recipients (7, 128). Similar to NK cells, DCs were described in organ transplantation with a double-edged role, since they can contribute both to rejection and tolerance. HLA-G molecules are known to exert immunosuppressive action on DC maturation and on NK cells, and can in consequence inhibit respectively T cell responses and NK cytolysis. Gros et al. showed HLA-G molecules impair NK/DC crosstalk *via* inhibition of dendritic cells (129).

So far, allograft rejection is a complex process involving both innate and adaptive immunity. Inflammation can contribute to rejection, and regulation of inflammation can reduce the incidence of rejection. Several studies refer to the role of HLA-G in inhibiting inflammation (130). Furthermore, rejection may be caused by antibodies against allo-antigens produced by B cells. HLA-G may have an inhibitory effect on B cells proliferation and differentiation thereby contributing to the graft acceptance post transplantation (131).

**Figure 3:**
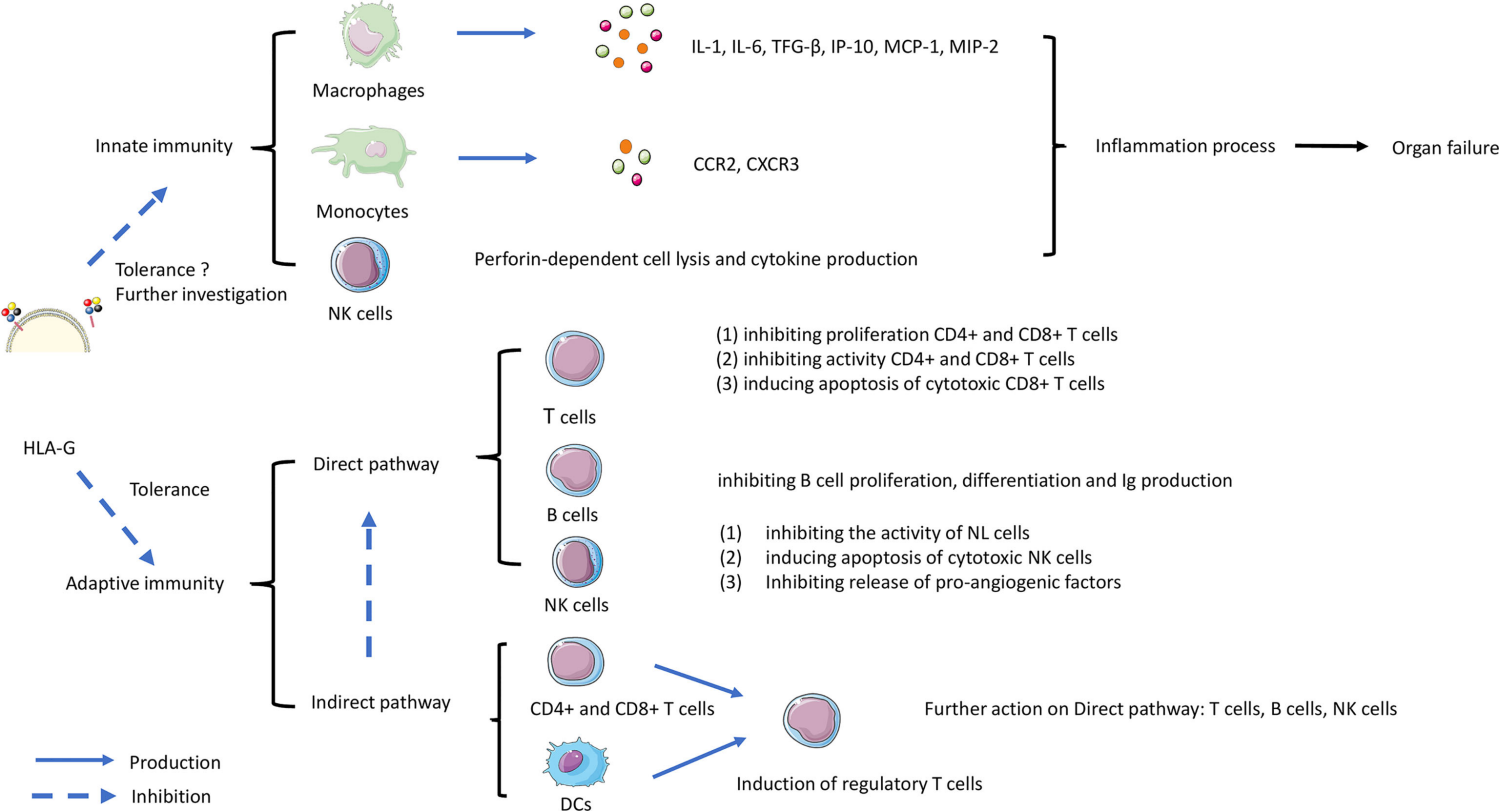
The role of HLA-G in controlling rejection in organ transplantation. The complex process involves both innate and adaptive immunity. HLA-G interaction on macrophages, monocytes, and NK cells can trigger the production of cytokines leading the inflammation contributes to rejection. Another interaction work on adaptive immunity involved direct and indirect pathways. The direct pathway is HLA-G directly inhibiting immune effectors such as T cells, B cells, and Natural Killer (NK) cells. The indirect pathway involves HLA-G acting on dendritic cells (DC) and CD8+/CD4+ T cells. Subsequently acting on T regulatory cell (T reg) formation, then continue acting on the direct pathway with inhibiting the functional cells.

**Figure 3** shows a schematic overview of the possible roles of HLA-G in mediating effects by direct and indirect pathways on T cells, B cells and DCs, which can contribute to immune tolerance. The complex process involves both innate and adaptive immunity. HLA-G interaction on macrophages, monocytes, and NK cells can inhibit the production of cytokines, that are involved in inflammation and rejection. The direct pathway is the interaction with ILT-2 and ILT-4 receptors on T cells, B cells and NK cells leading to inhibitory processes such as inhibition of proliferation, and inducing apoptosis of cytotoxic cells. Indirect effects of HLA-G may affect DCs, CD4+ and CD8+ T cells leading to induction of regulatory and suppressor T cells that will further act on effector cells. Although the role of the Fas/FasL system in allograft tolerance is not entirely clear, apoptosis of cytotoxic cells is an essential requirement for tolerance in transplantation (132). As discussed above HLA-G can induce CD8+ T cells apoptosis by interaction with FasL, but further research on this topic is needed.

### Relation of HLA-G With Immunosuppression Drugs or Other Risk Factors Post Transplantation

Immunosuppression is used to prevent rejection. Therefore it is appropriate to investigate the role of HLA-G regarding allograft status, and whether HLA-G can be used as a surrogate marker for monitoring rejection during immunosuppressive treatment.

Levels of sHLA-G in 17 heart transplantation recipients showed no relation to an immunosuppressive regimen treatment with cyclosporine (CsA). This study has the limitation of using serum samples and not plasma samples, while measuring sHLA-G was shown to be more accurate in plasma samples (133). Therefore, it is highly recommended to measure HLA-G in plasma because HLA-G maybe trapped in blood clots in serum (36). Higher sHLA-G expression was related to Everolimus (Eve) treatment but not to mycophenolate mofetil (MMF) treatment (133). Other investigations in kidney transplantation patients showed tacrolimus but not CsA to be associated with increased expression of HLA-G (90). *In vitro* studies showed that immunosuppressive drugs including everolimus, tacrolimus, cyclosporin, and dexamethasone could not induce HLA-G expression in human tubular epithelial cells (109). Lastly, no differences in sHLA-G levels were detected in liver transplantation patients without rejection after regular immunosuppressive therapy (116, 134). CTLA4-Ig (Belatacept) is a new recombinant molecule used for preventing acute rejection in kidney transplanted patients, and patients treated with CTLA4-Ig had higher sHLA-G plasma levels than patients treated with calcineurin inhibitors or healthy donors (135). Overall, these studies suggest that current immunosuppressive therapies may enhance expression of donor or recipient HLA-G or its receptors. These findings suggest that immunosuppressive therapy may affect sHLA-G concentrations post–transplant and that the expression of HLA-G confers protection against rejection.

### Prediction of Transplant Success by HLA-G Polymorphisms

In studies concerning the HLA-G polymorphisms, it has been shown that HLA-G polymorphism can act as a risk factor for diabetes mellitus in both kidney and pancreas transplantation (106, 123). Both HLA-G 14bp and +3142G/G show associations with obesity post kidney transplantation (92, 106). Low expression of HLA-G in patients with HLA-G 14bp was associated with dyslipidemia (93). HLA-G 14bp and 3187SNP were risk factors of cancer development post heart transplantation (136, 137). The frequency of HLA-G SNP -201 (CC) was increased in patients with cardiac allograft vasculopathy (CAV) after heart transplantation (138). In general, prediction of outcome can be based on polymorphism with changed expression of either sHLA-G or membrane HLA-G.

Infection is one of the main causes of death post transplantation. Renal transplant recipients with HLA-G +3142 CC genotypes had more CMV infections (108). A higher sHLA-G pretransplant was associated with more infections in heart transplantation (139). Also patients with CAV and high HLA-G expression were at risk for CMV post heart transplantation (140). Therefore HLA-G polymorphisms might also be a predictive marker in infection risks.

## Conclusion

In conclusion, the primary biological role of HLA-G to prevent the embryo from being rejected shows similarities to the process of transplantation. In this review we describe the roles of HLA-G during solid organ transplantation. In general we believe that the level of expression of HLA-G in donor tissues is positively correlated with graft acceptance by down regulation of the host immune response. Furthermore, it is clear that binding of HLA-G to its receptors on immune cells has a downregulatory effect on those immune cells. Measurement of soluble HLA-G in different compartments as an indirect measurement of immune activity is more difficult to interpret since it depends on the time line after transplantation and the dynamics of the involved immune cells in either acute or chronic rejection. Therefore, usage of sHLA-G as biomarker for evaluation of the course of transplantation or as predictor for acute or chronic rejection should be done with caution. HLA-G can also exert influence by inducing production of cytokines and by inducing differentiation to regulatory T cells. A few studies cannot confirm a role in graft acceptance, but show other associations between HLA-G and transplantation, such as a role in inducing inflammatory processes. Of note, some studies show that immunosuppressive treatment such as the use of everolimus, tacrolimus and belatacept could upregulate HLA-G expression, thereby inducing a more stable environment for the graft. It is important to keep in mind that polymorphisms in HLA-G can predict variation in HLA-G expression and therefore can play a role in transplantation rejection. Recent findings indicate also a relation between HLA-G expression and inflammation, which is another risk factor for rejection post transplantation. Future studies may focus on the multiple working mechanisms of HLA-G in immune regulation and how that knowledge can best be used in maintaining a balance between prevention of rejection and maintaining a adequate immune system after transplantation.

## Author Contributions

SL, JW, DB and NB contributed to the conception, design, and writing. EV, JW, NB, and DB contributed to the revision of this article. All authors contributed to the article and approved the submitted version.

## Funding

This work was financially supported by the Chinese Scholarship Council (CSC NO.201909110111).

## Conflict of Interest

The authors declare that the research was conducted in the absence of any commercial or financial relationships that could be construed as a potential conflict of interest.

## Publisher’s Note

All claims expressed in this article are solely those of the authors and do not necessarily represent those of their affiliated organizations, or those of the publisher, the editors and the reviewers. Any product that may be evaluated in this article, or claim that may be made by its manufacturer, is not guaranteed or endorsed by the publisher.

## Abbreviation

HLA-G, Human Leukocyte Antigen-G; MHC, Major histocompatibility complex; β2M, β-2-microglobulin; UTR, Untranslated region; URR, Upstream regulatory region; PBMCs, Peripheral blood mononuclear cells; SNPs, Single-nucleotide polymorphisms; NK, Natural killer; DC, Dendritic cells; TBBx, Systematic transbronchial biopsies; AR, Acute rejection; ESRD, End-stage renal disease; MMF, Mycophenolate mofetil; RAD, Everolimus; BOS, Bronchiolitis obliterans syndrome; AMR, Antibody mediated rejection; hc, Heavy chain; ILT, Ig-like transcript; KIR, Killer cell Ig-like receptor; LILR, Leukocyte immunoglobulin-like receptor; TGF-β, Transforming growth factors; CsA, Cyclosporine; IFN, Interferon; APCs, Antigen-presenting cells; SOT, Solid organ transplantation.
