# Supplementary material for: Biological Characteristics of HLA-G and Its Role in Solid Organ Transplantation
Source: Front Immunol. 2022 Jun 13;13:902093. doi: 10.3389/fimmu.2022.902093 (PMC9234285; doi:10.3389/fimmu.2022.902093)
Supplement: Supplementary file 1 [file Table_1.docx]

Table S1. 3’ UTR haplotypes of the HLA-G locus and the HLA-G alleles associated with each haplotype

| **HLA-G haplotypes** | **HLA-G 3’ UTR** | | | | | | | | **HLA-G allele** |
| --- | --- | --- | --- | --- | --- | --- | --- | --- | --- |
|  | **14bp** | **+3003** | **+3010** | **+3027** | **+3035** | **+3142** | **+3187** | **+3196** |  |
| UTR-1 | Del | T | G | C | C | C | G | C | G*01010101/02 |
| UTR-2 | Ins | T | C | C | C | G | A | G | G*01010201  G*010114  G*0105N  G*0106^c^  G*0109 |
| UTR-3 | Del | T | C | C | C | G | A | C | G*010401  G*010403  G*010404 |
| UTR-4 | Del | C | G | C | C | C | A | C | G*01010105^d^  G*010109 |
| UTR-5 | Ins | T | C | C | T | G | A | C | G*010108  G*0103 |
| UTR-6 | Del | T | G | C | C | C | A | C | G*01010101/02  G*01010104  G*01010105^d^ |
| UTR-7 | Ins | T | C | A | T | G | A | C | G*010103  G*010105 |
| UTR-8 | Ins | T | G | C | C | G | A | G | G*0106^c^ |
