# Supplementary material for: Biological Characteristics of HLA-G and Its Role in Solid Organ Transplantation
Source: Front Immunol. 2022 Jun 13;13:902093. doi: 10.3389/fimmu.2022.902093 (PMC9234285; doi:10.3389/fimmu.2022.902093)
Supplement: Supplementary file 2 [file Table_2.docx]

Table. S2. HLA-G receptors on immune cells and function.

| Cells | Receptors | Function |
| --- | --- | --- |
| Mast cells | KIR2DL4 | Inhibit cell mediated allergic reactions |
| T cells | ILT-2, CD8 | Inhibition of proliferation  Inhibition of cytolysis  Induction of Tregs  Induction of Th2-type cytokine  Inhibition of chemotaxis  Inhibition of proliferation, cytotoxicity |
| B cells | ILT-2 | Inhibiting of proliferation  Inhibiting of differentiation  Inhibiting of Ig production |
| Monocytes | ILT-2, ILT-4 | Inhibiting function |
| Endothelial cells | CD160 | Inhibiting angiogenesis |
| Macrophages | ILT-2, ILT-4 | Inhibiting activation |
| NK cells | ILT-2, ILT-4, CD8, KIR2DL4 | Inhibition of cytotoxicity  Inhibition of IFN-γ secretion  Inhibition of chemotaxis |
| Dendritic cells | ILT-2, ILT-4 | Induction of tolerogenic DC  Induction of suppressor T cells  Inhibition of NK cell activation |
